# Coverage, social mobilization and challenges of mass Zithromax administration campaign in South and South East zones of Tigray, Northern Ethiopia: A cross sectional study

**DOI:** 10.1371/journal.pntd.0006288

**Published:** 2018-02-26

**Authors:** Afework Mulugeta, Gebremedhin Berhe Gebregergs, Selamawit Asfaw, Dejen Yemane, Mengistu Mitiku, Beyene Meresa, Goitom Gigar, Amanuel Kidane

**Affiliations:** 1 School of Public Health, College of Health Sciences, Mekelle University, Mekelle, Ethiopia; 2 Tigray Regional Health Bureau, Mekelle, Ethiopia; RTI International, UNITED STATES

## Abstract

**Background:**

The antibiotic treatment of people with trachoma helps to prevent transmission the disease in a community. Currently, Zithromax is the drug recommended for mass drug administration (MDA). MDA should be carried out annually for three to five years in trachoma endemic areas. Coverage survey is essential to track progress towards program goals and to identify communities with poor coverage in order to permit timely and appropriate actions. We assessed mass Zithromax administration coverage, social mobilization and campaign challenges in south and southeast zones of Tigray, Ethiopia.

**Method:**

We conducted a survey in community in Southern and South East zones of Tigray region from August 15 to August 31, 2016. The survey included nine Woredas. It was supported by qualitative methods. A total of 3741 individuals were enrolled from 933 households using multistage sampling. We used structured questionnaire. In-depth interview and focus group discussion were also applied. Descriptive statistics was performed using SPSS version 20.We thematically analyzed the qualitative data using Atlas 7.

**Result:**

The overall coverage of Zithromax MDA was 93.3%. It ranges from 90.0% in Seharti Samre to 97.9% in Endamokoni. The coverage was 93.4% for males and 93.1% for females. A higher proportion (98.3%) of children aged 5 to 15 years and 409 (87.8%) under five children took Zithromax. The coverage was 94% in rural and 91.2% in urban. Women development army (43.3%) and health extension workers (32.5%) were the main source of information. Frequent occurrence of drug side effects, rumors, lack of community and leaders’ engagement in the campaign, fasting, shortage of human power and short term unavailability of supplies were barriers during the campaign.

**Conclusion:**

The Zithromax MDA coverage in the study zones was higher than the minimum WHO set criteria of 80%. There was a wide difference in coverage among Woredas and Kebeles. The MDA coverage was lower in urban than rural. Misconceptions and poor mobilization were common challenges. Thus, proper planning, community mobilization and uniform training will need to be done ahead of the campaign in the future.

## Author summary

Ethiopia has the world's highest burden of trachoma. The disease causes significant blindness and visual impairment, particularly in poorer communities. To prevent the transmission of active trachoma, mass administration of Zithromax is given every year, which is implemented for consecutive three to five years in endemic and hyper endemic areas of the country. We have shown that the mass administration coverage of Zithromax, in 2016, was greater than 90% in the South and Southeastern zones of Tigray, Ethiopia. However, it was accompanied by a number of program challenges and social barriers.Women development armies and health extension workers were key players in community mobilization and drug provision, respectively.

## Introduction

Globally, trachoma is the leading infectious cause of blindness and one of the neglected tropical diseases [1]. According to World Health Organization (WHO) report 2016, trachoma is responsible for 3% of global blindness and 2.2 million people with visual impairment. It blinds one person every 15 minutes and makes one person experience severe sight loss every four minutes. Fifty three countries are estimated to be endemic for blinding trachoma and around 200 million people live in those areas [2,3].

Trachoma is more prevalent in low and middle income countries. Africa is the most affected continent with 27.8 million cases of active trachoma and 3.8 million cases of trichiasis which makes up 68.5% and 46.6 of global estimates, respectively[4]. Ethiopia has the world's highest burden of trachoma; with 75 million people at risk of active infection. It along with India, Nigeria, Sudan and Guinea contribute 48.5% of the global burden of active trachoma, with about half of the global burden concentrated in three countries: China, Ethiopia and Sudan [5, 6].

In 1997, World Health Organization established an ‘Alliance for Global Elimination of Trachoma by the year 2020’. For its successful implementation, WHO endorsed an integrated package of interventions known as SAFE, an acronym which stands for Surgery for Trachomatous trichiasis, Antibiotic treatment for ocular *Chlamydia trachomatis* infection, Facial cleanliness to reduce the transmission of the infection and Environmental improvement focusing on improved access to water and sanitation. Trachoma control program implementation is prioritized in communities where prevalence of active trachoma in children aged 1 to 9 years is 10% or higher and/or where the prevalence of TT in people aged 15 or above is one percent or higher. One round treatment is also recommended in areas between 5% - 9.9% infection in children aged under 10 years [3,7–8].

Currently, Zithromax is the drug recommended for mass drug administration (MDA), with the exception of children < 6 months of age for whom tetracycline eye ointment (TTC) should be given [9]. MDA should be carried out annually for three years which can extend up to five years in hyper endemic areas before a repeat prevalence survey is conducted [2,10]. In March 2002, Zithromax was registered in Ethiopia for the management of trachoma [5]

Following the launch of Vision2020 [1], Ethiopia established a national steering committee for trachoma control in 2001 and launched ‘Vision2020 initiative’ in September 2002. By the end of 2012, the country took part in the Global trachoma mapping. Ethiopia has completed trachoma mapping and nationwide a total 726 districts are mapped for trachoma. These districts have trachoma of public health significance despite many efforts are made so far to address the problem[11,12].

Tigray is one of the regions in Ethiopia which is highly hit by trachoma. In this region, 34 districts and six sub districts are mapped for trachoma [13]. These cover over three quarters (76.9%) of the districts in the region. Tigray Regional Health Bureau and LIGHT FOR THE WORLD are working together to eliminate blinding trachoma in Tigray, Ethiopia by implementing the WHO recommended SAFE strategy. The Zithromax mass administration had started in Southern and South east zones of Tigray in nine rural districts in 2014.These zones have highest trachoma prevalence in the region. The first round of mass administration of Zithromax was conducted in 2014; the second and third round was conducted in June 2015 and May 2016, respectively.

Immediate coverage survey is essential to track progress towards program goals and to identify communities with poor or insufficient coverage in order to permit timely and appropriate actions to improve coverage. Besides, it helps to identify common reasons for not swallowing the drugs and program managers can improve social mobilization prior to the next MDA round. Thus, in this survey, we assessed the coverage of Zithromax mass administration in selected districts in which the program was instituted. In parallel, we explored the program challenges and social barriers during mobilization and drug administration in the study zones.

## Method

### Study setting

**Fig 1:**
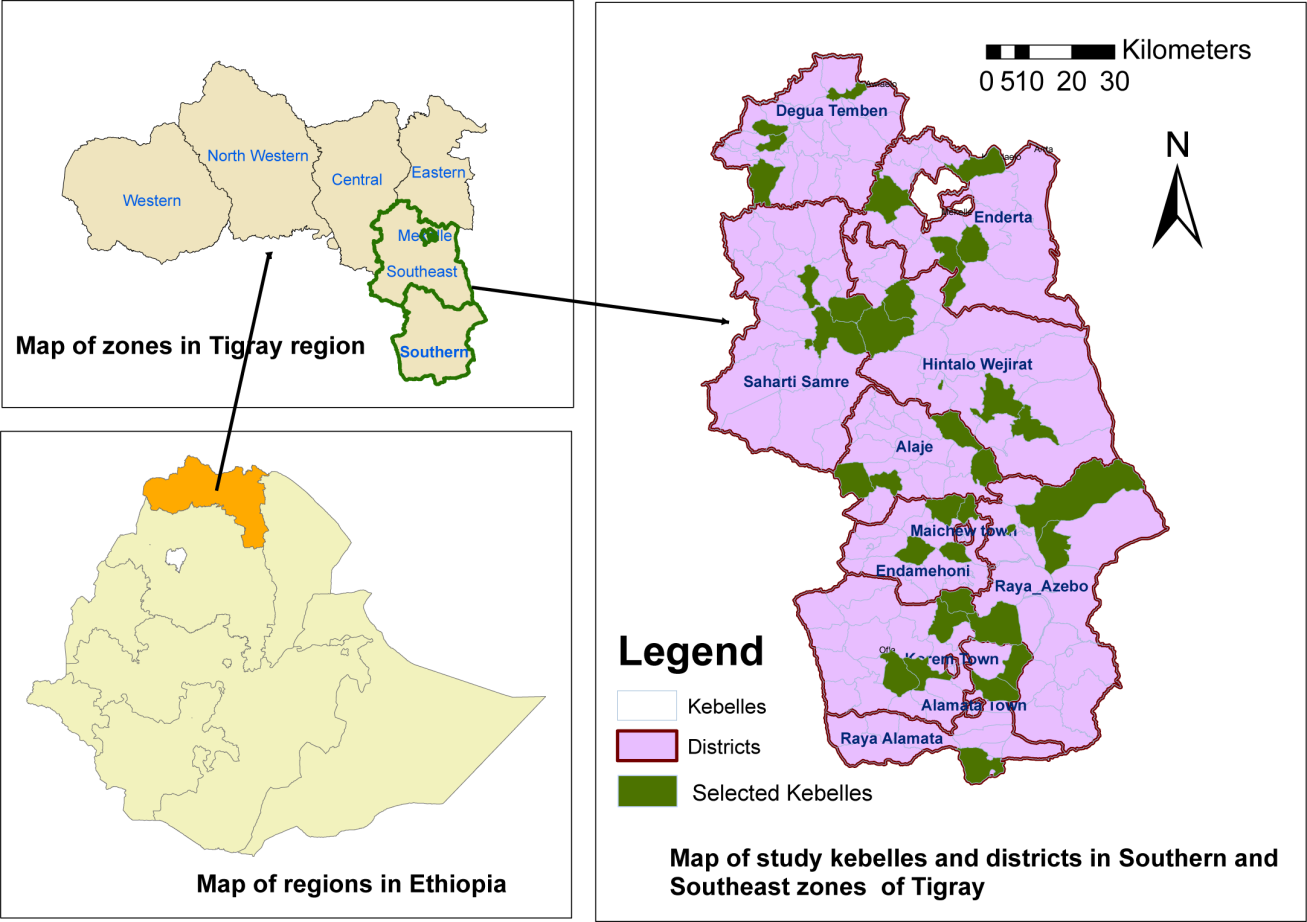
Geographical map of study districts and kebelles in South and Southeastern zones of Tigray region, 2016.

The survey was conducted in Southern and South East zones of Tigray region, Ethiopia. There are four Woredas in South East and eight Woredas in Southern zone (Fig 1). According to the 2007 census, the estimated population was 392,142 in South East and 1,006,504 in Sothern zone. The Zithromax MDA campaign was carried out in June 2015 (second round) and in May 2016 (third round) in both zones.

### Study design and participants

We conducted an MDA coverage survey in community from August 15 to August 31, 2016. To strengthen the study, we explored barriers to Zithromax uptake during the campaign. We included all family members who have been in the time of the campaign (May 2016) in the selected households from the two zones. Children who were less than 6 months and pregnant women in the first trimester during the 2016 MDA program were excluded from the study. District health experts and Health Extension Workers (HEWs) were key informants. Likewise, women development armies and community representatives were focus group discussion (FGD) participants.

### Sample size calculation and procedures

We determined sample size of 937 households using single population proportion formula. Assumptions: MDA coverage of 77% [14], 95% confidence level and 4.25% degree of precision. We used design effect of 2 and added 10% non-response rate. Multi stage with systematic random sampling was used. Nine districts, where trachoma MDA was carried out, were purposefully included in the study. In each district, kebeles (the smallest admistrative unit) were selected by lottery method. Finally, households in the selected kebele were enrolled using systematic random sampling technique.

In qualitative part, two key informants were purposively selected in each district using four criteria: 1) involvement in the whole process of campaign, 2) frequent contact with the community, 3) exposure to the management process of the campaigns and 4) taking part in the consecutive Zithromax campaigns in the Woreda. Likewise, one FGD was conducted in each of the nine Woredas. Each FGD consisted of 8 to 10 participants.

### Data collection procedure

#### Quantitative data

investigators used structured and pre tested questionnaire. We collected socio demographic information, Zithromax uptake, type and source of health information. Twelve health professionals had collected the data. We interviewed heads of household about himself, children and about family members who are absent during the data collection. Other adults in the household were asked independently if they had been offered and swallowed the drug. To decrease recall bias, the team had physically showed the drug to respondents.

#### Qualitative data

our team developed semi structured FGD and in-depth interview guides. The FGD guide was designed to capture the following issues: community perception, acceptability of Zithromax mass administration, social mobilization and challenges during the campaign. The in-depth interview guide mainly designed to grasp: how the Zithromax mass treatment campaign was going on, success stories of the mass Zithromax treatment coverage for each campaign and the challenges during the drug distribution.

### Data quality control

We pre-tested the questionnaire and translated it into Tigrigna. Data collectors and supervisors were trained for two days on how to properly fill the questionnaire, data collection techniques and the purpose of the study. During data collection, the investigators took field notes and independently audio taped the interviews and FGDs. The study team supervised the process on a daily basis. We reviewed and checked the questionnaire for completeness, accuracy and consistency.

### Data processing and analysis

Data were entered into the Epi- data and analyzed using SPSS version 20. Descriptive statistics like the survey coverage was calculated. The coverage was further stratified by age, sex and district. For qualitative data: all independently recorded FGDs and in-depth interviews were transcribed and translated word by word at each step after a repeated listening. We imported, translated documents to Atlas version 7 for analysis. We coded respondent’s words, phrases and sentences. The selected codes were synthesized in families, and finally non-repetitive themes were developed.

### Ethical considerations

We received approval for this research from an ethical committee or institutional review board of Mekelle University, college of health sciences. Support letter was obtained from the Tigray regional health bureau and permission was sought from each selected Woreda health office and respective kebele. Informed verbal consent was obtained from participants after they get full information on the purpose of the study. Anyone who was not willing to take part in the study was excluded. Finally the study did not disclose any information by the name of participants to assure confidentiality. During analysis, all data were anonymized.

## Result

### Quantitative findings

#### Socio -demographic characteristics of respondents

A total of 3741 individuals were enrolled from 933 households across two zones of Tigray in the post 2016 MDA survey. Participants had an average family size of 4.1. Southern zone contributed 60% of the total sample. Female participants were 1962 (52.5%) and rural residents comprise 2800 (74.8%). Regarding age distribution, 2132 (57%) individuals were aged greater than 15 years and 466 (12.5%) were aged less than 5 years. About half of the study participants were married. One third and one fourth of the study subjects were students and farmers, respectively. One thousand seventy two (28.9%) study participants were unable to read and write (Table 1).

**Table 1:** Socio-demographic characteristics of respondents in South and Southeastern zones of Tigray, 2016.

Variable	Category	Frequency	Percentage
**Zone**	South	2245	60.0
	Southeastern	1496	40.0
**Residence**	Rural	2800	74.5
	Urban	941	25.5
**Sex**	Female	1962	52.5
	Male	1778	47.5
**Age**	<5 year	466	12.5
	5–15 year	1143	30.6
	>15	2132	57.0
**Marital status(n = 2651)**	Married	1313	49.5
	Single	1067	40.0
	Divorced	199	7.5
	Widowed	72	3.0
**Educational status(n = 3163)**	Unable to read and write	1072	28.7
	Able to read and write	132	3.5
	1–4 grade completed	617	16.5
	5–8 grade completed	674	18.0
	9–12 grade completed	553	14.8
	College diploma	65	1.7
	College degree	50	1.3
**Occupation(n = 3101)**	Student	1236	33.0
	Farmer	948	25.3
	Merchant	286	7.6
	House wife	222	5.9
	Unemployed	211	5.6
	Government employee	106	2.8
	NGO	48	1.3
	Others*	44	1.2

* Daily laborer, driver, hair dresser, miller and sex work

#### Zithromax mass drug administration coverage

**Fig 2:**
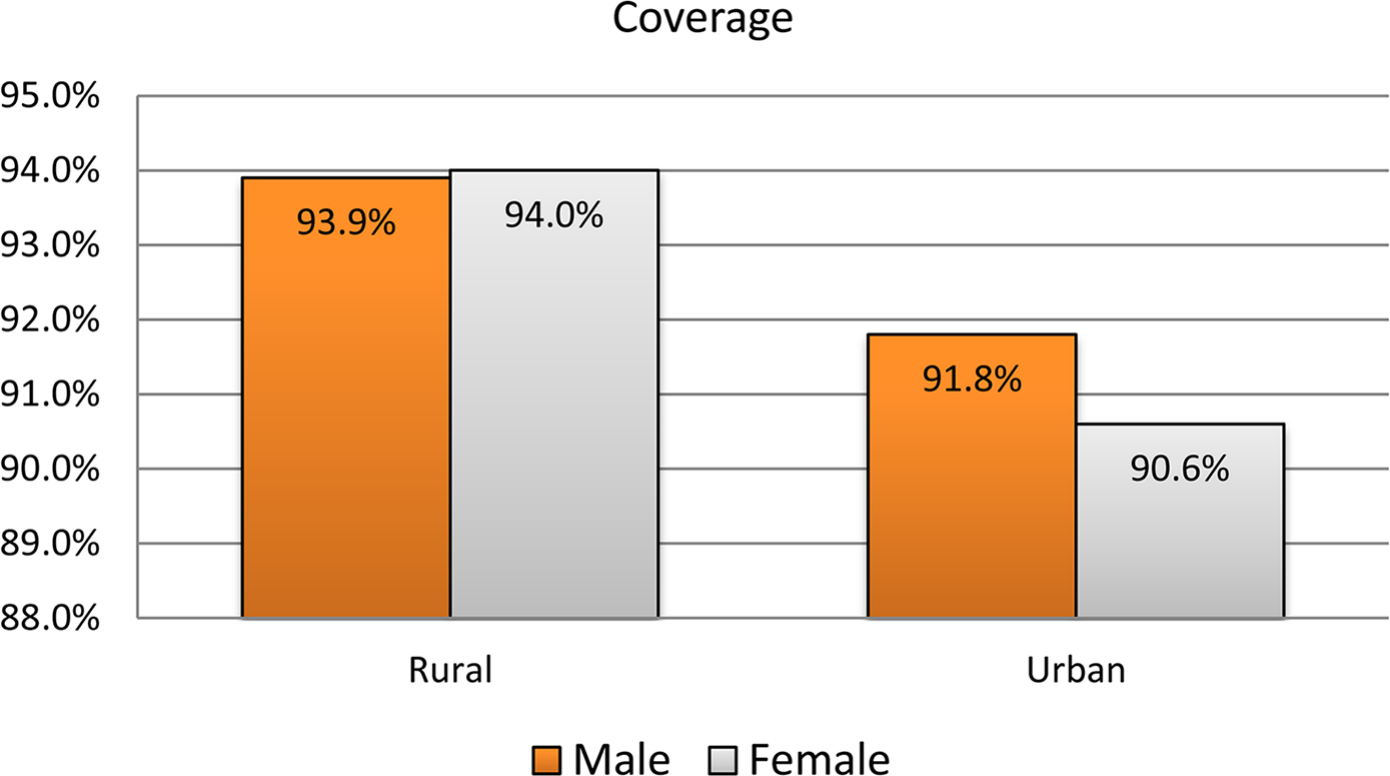
Zithromax coverage stratified by sex and residence in South and Southeastern zones of Tigray region, 2016.

**Fig 3:**
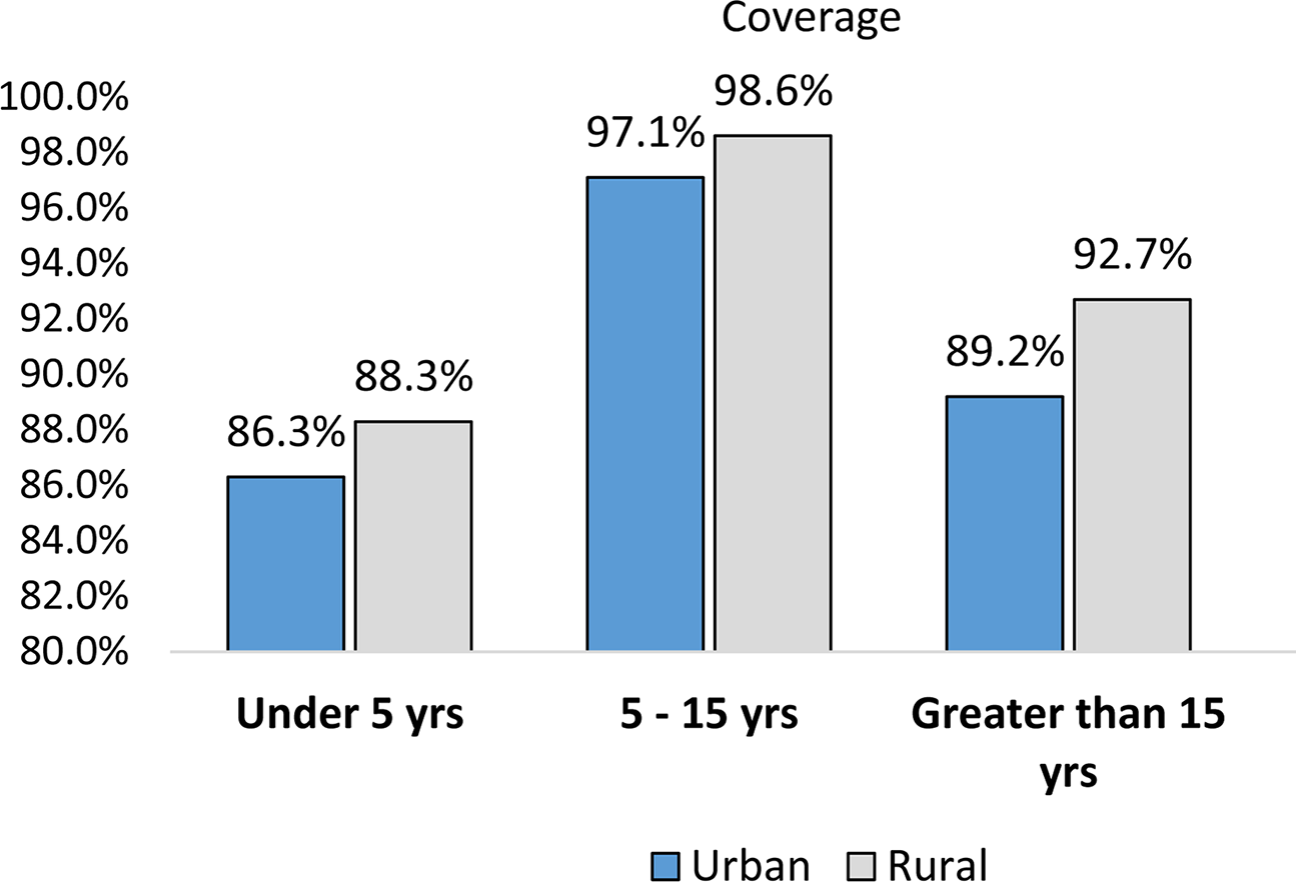
Zithromax coverage stratified by age and residence in South and Southeastern zones of Tigray region, 2016.

The overall coverage of Zithromax mass drug administration in the recent campaign (May 2016) in the study zones (South and Southeastern) was 93.3% (3489 /3741) with 95% CI: 92.4% -94.0%. With regard to sex, 1661 (93.4%) males and 1828 (93.1%) females had received Zithromax. From the age eligible targets, a higher proportion (98.3%) of children aged 5 to 15 years and 409(87.8%) under-five ones took Zithromax. Nearly the same coverage of MDA was observed in both zones (Table 2). The coverage was better in rural than urban community and the difference was statistically significant (p-value = 0.003).When the coverage is stratified by residence and adjusted for age and sex separately, still the coverage was better in rural than urban (Figs 2 and 3).

**Table 2:** Mass drug administration coverage by selected variables in South and Southeastern zones of Tigray, 2016 (N = 3741).

Variables	Category	Zithromax received		Total
		Yes, n (%)	No, n (%)	
**Sex**	Male	1661(93.4)	117(6.6)	1778
	Female	1828(93.1)	135(6.9)	1963
**Age**	Less than five years	409(87.8)	57(12.2)	466
	5–15 years	1123(98.3)	20(1.3)	1143
	Greater than 15 years	1957(91.8)	175(8.2)	2132
**Residence**	Rural	2631(94.0)	169(6.0)	2800
	Urban	858(91.2)	83(8.8)	941
**Zone**	Southern Tigray	2097(93.4)	148(6.6)	2245
	Southeastern Tigray	1392(93.0)	104(7.0)	1496

**Fig 4:**
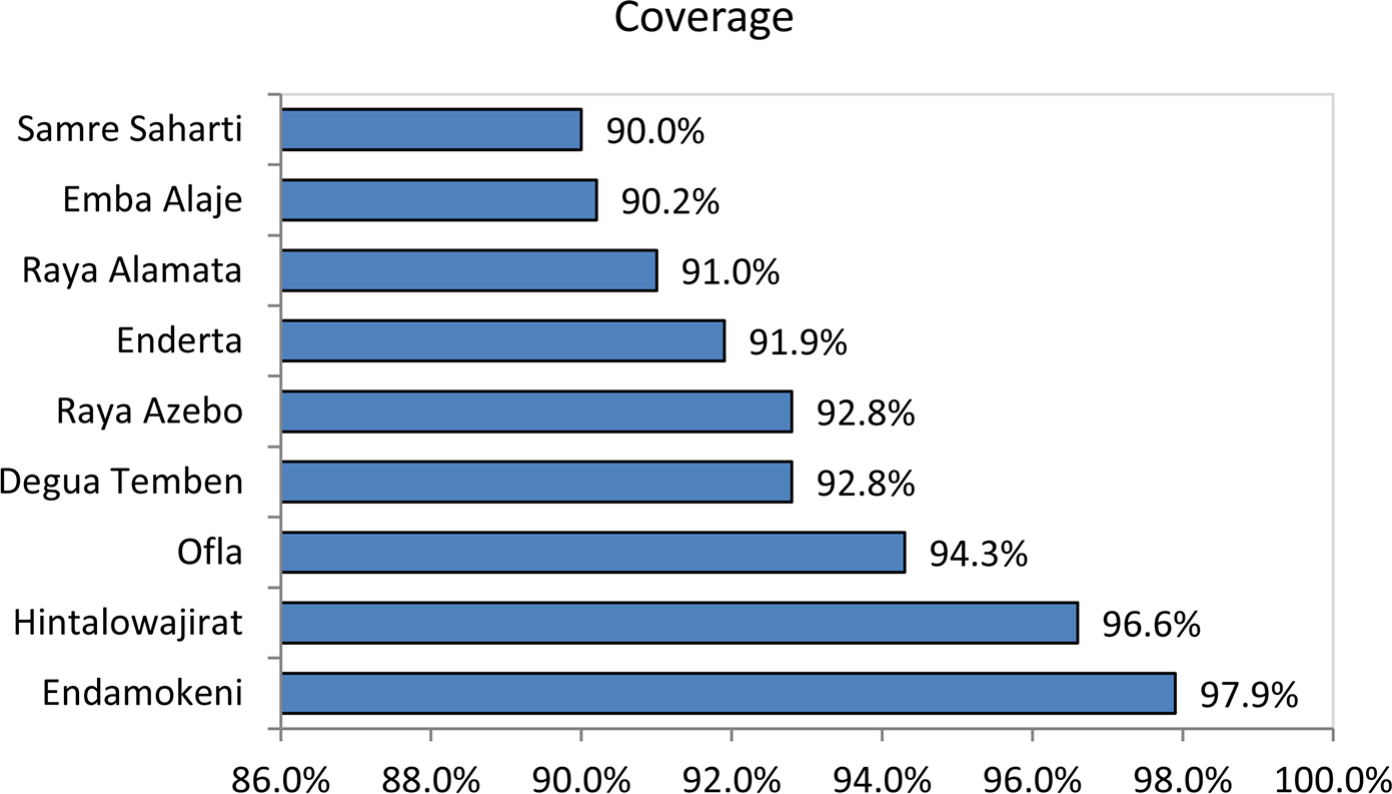
Zithromax MDA coverage by district, Southern and Southeast Zones of Tigray, 2016.

Zithromax coverage ranges from 90.0% in Seharti Samre to 97.9% in Endamokoni District (Fig 4). In the study Kebeles, it was as low as 75.4% in Didiba of Enderta District and as high as 100% in Senay of Endamokoni District.

#### Type and sources of information about Zithromax mass administration

Among those who received Zithromax mass treatment, 850 (92.7%) were informed about the campaign ahead of time; of which 481(43.3%) and 361(32.5%) got the information from women development armies and health extension workers, respectively.

Of the total that swallowed the drug, 478(52.70%) study participants got health information during the campaign. The three main health messages given were: Zithromax prevents from contacting eye disease (22.2%), personal hygiene is critical to prevent from contracting eye diseases (20.0%) and advice to take the ‘drug’ (18.1%); Table 3.

**Table 3:** Type and source of information about Zithromax mass administration in Southern and Southeast zones of Tigray, 2016.

Variables	Category	Frequency	Percentage
**Get informed about the campaign**	Yes	850	92.7
	No	67	7.3
**Source of information**	Women development army	481	43.3
	Health extension worker	361	32.5
	Kebele leaders	96	8.6
	Social mobilizers/Public criers	76	6.8
	Other health professionals	66	5.9
	Religious leaders	23	2.1
	TV	4	0.4
	Community leaders	2	0.2
	Radio	1	0.1
**Health information given by HEW**	Yes	478	52.7
	No	429	47.3
**Type of information given**	Personal hygiene	129	20.0
	Drug prevents from eye disease	143	22.2
	Advice to take the drug	117	18.1
	Wash your face	64	9.9
	Environmental sanitation	48	7.4
	Wash your hands	47	7.3
	Take food before the drug	31	4.8
	Trachoma causes blindness	18	2.8
	Pregnant mothers do not take the drug	13	2.0
	Take appropriate dose of drug	11	1.7
	Get vaccination	11	1.7
	Food hygiene	5	0.8
	Do not drink alcohol	5	0.8
	Get adequate rest	3	0.5

#### Reasons for non-participation on mass drug administration

Two hundred nineteen individuals (6.7%) didn’t receive Zithromax. The main reasons were ‘absence’ during campaign 104(47.5%), wrong mothers’ perception on eligibility of their child 36 (16.4%) and lack of awareness about the campaign 20(9.1%); Table 4.

**Table 4:** Reasons for non-participants on Zithromax MDA in Southern and Southeast zones of Tigray, 2016.

Reasons for not receiving(n = 219)	Frequency	Percent
Absence	104	47.5
Perceived the child is not eligible	36	16.4
Not aware	20	9.1
Others*	18	8.2
Pregnant	16	7.3
S/he was in school	10	4.6
Refusal	9	4.1
The house wasn’t visited	6	2.7

*being sick, working at home, taking other drug

### Qualitative findings

#### Theme I: Pre MDA campaign activity: Social mobilization

According to the respondents, there was social mobilization in all Woredas before the campaigns. The duration of the mobilization ranges from three days to two weeks. The effort and time given for mobilization were shorter in 2016. In some kebeles, it was poor and lengthy. Despite these limitations, political and religious leaders, health workers, women development army and community representatives had involved in the dissemination of information about the MDA campaign. A key informant from Raya Alameda indicated that "….. *there were no concerted efforts of mobilization in the 2016 campaign*. *Consequently*, *the campaign took up to ten days to be completed" and we feel tired*. In addition to this, a campaign on soil and water conservation was arranged on the same period. Approaching the community members involved in the conservation activity was challenging and lengthy. However, drug providers had shown their commitment to access the hard to reach areas and use house-to-house administration of the drug to reach the older people who cannot come to the station.

A 32 year old women FGD participant revealed that:

"….. *The station for the campaign was not convenient for elderly people and it was too far from their home*. *Hence*, *I recommend future campaigns to be as close as possible to Kushet level (the lowest administrative unit)"*.

Although key informants mentioned ‘a coordination of the sectors’ in Raya Alamata during the campaign, the health extension workers, health development armies and the community representatives in the FGD revealed that most of the kebele leaders were engaged in other activities that makes hard to access the household members of the sites for the campaign.

#### Theme II: Intra-campaign events

Despite the timely distribution of supplies and proper mobilization of health professionals as claimed by the Woreda, there were minor problems regarding this issue. Participants reported that there was a delay in distribution of registration books in Ofla Woreda, shortage of TTC ointment till the third day of the campaign in Raya Alamata, and shortage of the drug in Seharti Samre and Raya Azebo.

According to the respondents, mass administration of Zithromax was delivered at *“Got” level/“Kushet”* and at health facilities (health centers and health posts) and schools. Schools were convenient places for students. A Key informant from Raya Alamata also indicated that providing the intervention at schools in the first days of the intervention helped them for three purposes. "….. *First*, *it avoids double dosing of individuals*. *Secondly*, *it helped as part of mobilization in the whole community because students would tell their parents at home*. *Finally*, *it reduced the burden in the community in the provision of the drug because large segment of the population (students) would be reached at the least cost for mobilization and transportation*…..*”*

In the second round (2016) campaign, most of the Woredas apply mass administration at Got” level, and it was the place where convenient to most of the community members. Furthermore, a problem which is frequently reported was mobilizing the whole population at the same day and at the same place for the mass administration of the drugs. Thus, a large number of the population came to the intervention sites and posed disproportionate burden on the providers to administer and record. Thus, the service users would grumble for the possible long waiting hours to get the drugs.

Participants explained the situation as follows. *“In 2015*, *we mobilized the whole community of the “Kushet” and faced problems*. *The first problem was that we were not able to identify who had and had not taken the drug on a daily basis that made our monitoring process too difficult*. *Secondly*, *a lot of people came together at the same time making the task unmanageable to counsel*, *administer and register*. *In the 2016 campaign*, *we took lessons from the past and we mobilize at “Got or village” level with a specific plan of the day and place for intervention*. *This had avoided the long waiting hours for the beneficiaries*. *Hence*, *the approach was convenient and acceptable to the community*.*”*

In the 2016 campaign, the role of the women development armies was repeatedly appreciated by the respondents. They were helpful for the orderly and timely mobilization of the community to the drug administration sites. Each leader of women development armies was responsible to mobilize and send 30 to 50 army members a time in an orderly fashion. This had greatly facilitated the smooth provision of the service and helped providers to manage the flow, registration, monitor the unreached members and to move to the next “Got” accordingly.

#### Theme III: Drug administration

“The type of drug and the number of pills an individual is required to take should be determined by age” as suggested by the community FGD participants. However, in case of children, height was used to determine the type of drug and the number of pills. According to the respondents, there were more than two types of measurement tools with visible variation in length. Few participants suspected that the tool was prone to systematic error. They suggested ‘age’ to determine the number of pills of zithromax rather than height.

Due to the side effects of the Zithromax in the 2015, ambivalence to take the drug and the hesitation of its benefits were common scenarios in the early days of the campaign. They were waiting until they see someone taking similar drugs. Consequently, the providers used role models in their respective sites to inspire the community to take the drug. The role of the Woredas administration has been impressive in convincing the community members and increases the uptake of the drug during the campaign.

*"*….. *Our participation was very important*. *I firstly took the drug to encourage those who came to the station and we informed them about the possible side effects….”* curative and rehabilitation expert of district health office in Hintalo Wajerat.

#### Theme IV: Drug side effects

Most of the FGD participants in the current study reported that the side effects of the drug in the 2016 campaign were remarkably lower compared to the 2015 campaign. However, few FGD participants reported that the drug in the 2016 campaign was heavier than the former one; with stronger side effects. In triangulation of this finding, pattern analysis of responses of Woreda key informants revealed that the difference was not in the drug, but in the conditions related to taking the drug after meals, with water and the presence of another illness at the time of the campaign.

FGD participant in Hintalo Woreda explained it the situation as

“In the first days of the campaign, there was a fear of side effect among the community members and there were inclinations to avoid the drug. However, after awareness creation the misconceptions and rumors were clarified and people start to understand the reality and take the drug”.

#### Theme V: Challenges of the campaign

Respondents repeatedly mentioned that the time of both campaigns was inconvenient to most of the people in the study sites. The campaign was conducted at the end of May, 2016. May is considered as a critical time for farming, and fasting. This month is characterized by hot weather which makes it difficult to walk to the drug administration sites during the day time especially at noon.

In the current campaign, youngsters were resistant and refused the providers. According to the participants, young people (some specified it as less than 24/25 years) resisted to take the drugs frequently compared to their older counterparts. The explored reasons were low perceived risk of Trachoma, lack of the awareness on the benefits and over exaggeration of possible side-effects. Moreover, there were misconceptions on the drug: ‘medication is only for those who actually have the disease’ and the desire to be autonomous in decision-making. Different strategies like counseling, clarifying rumors and explaining the benefits of the drugs were used to convince the male youngsters.

Head of district health office in Alamata described “a 58 years woman who had chronic disease was given the drug while she should be excluded from the campaign. She died within four hours after she took the drug.” Thus, it endangered the acceptance of the drug for a while. It raises refusal and rumors that expanded to other sites within and out of the Woreda in a short period of time.

Rumors about the drug and the delay in the mobilization of the supply and human power were among the critical challenges. In addition, diarrhea had been the most frequently reported side effect by the respondents. The participants reported that individuals had experienced diarrhea that lasts for a minimum of two/ three days to two months after the campaign.

Key informants in Raya Alamata reported that there was a de-worming campaign among under-five children two weeks ahead of Zithromax MDA. That had its own side effects and the key informant put his fear like this: repeated campaigns with short time intervals could create community fatigue.

In Raya Alamata some community members perceived that Zithromax harms reproductive organs and decreases sexual feeling. The key informants added that following the orientation not to use the ‘pill form of Zithromax’ for pregnant women with first trimester, a rumor began to circulate in the community to associate the death of a pregnant woman to the drug. This has further led to the emergence of another rumor which is Zithromax a contraceptive pill or family planning methods. These rumors were widely reported by FGD participants from Raya Azebo too. Concerns related to drug expiry were also echoed by the youngsters from Alaje Woreda.

One religious leader from Hintale Wajirat opposed the campaign. He asserted that *“*….. *We should only rely on God rather than such governmental health services including immunization*…..”

Both campaigns were affected by fasting days. Respondents mentioned that Muslim communities were fasting during the campaign in 2016. This makes the provider to wait up until evening to administer the drugs at their home. The participants also reported that Christians who fasted took the drugs before meal in the 2015. This was associated with heavier side effects. On Wednesday and Friday, fasting days, elders were waiting until noon to swallow the drug.

## Discussion

### Zithromax mass administration coverage

The overall coverage of Zithromax mass drug administration in the recent campaign (May 2016) in the south and southeastern zone was found to be 93.3%. This is higher than the minimum coverage rate of 80% recommended by WHO [15]. When a coverage is greater than 90%, there will be a lower chance of trachoma recurrence until the next round of annual mass drug administration is underway [16]. This coverage is also higher than a study conducted in Northern Tanzania which was 76% in 2005 and 76.9% in 2011 [17]. Another study conducted in Nigeria in 2013 reported a coverage of 60.3% [18]. The coverage in our survey is also higher than studies conducted in other parts of Ethiopia: Goncha Siso Woreda and Injibra town which was 88.8% and 92.2% respectively [14,19]. The higher coverage rate in this survey might be because it is a recent study and Zithromax drug administration might be getting higher acceptability with time. In addition, this might also be because of the great effort put to mobilize and increase access to information as it was reflected from the qualitative study.

There was statistically significant difference in coverage between rural (94%) and urban (91.2%). A similar result was found in a study conducted in Awi zone, Amhara region: 94.3% in rural and 89.2% in urban residence[14]. This difference might be due to resistance that usually observed in urban communities.

There was a significant difference in the MDA coverage between woredas and between kebeles. Zithromax coverage ranges from 90.0% in Seharti Samre woreda to 97.9% in Endamokoni woreda. Among the study Kebeles, the coverage was also varied. It was as low as 75.4% in Didiba of Enderta Woreda and as high as 100% in Senay of Endamokoni woreda. These variations might be explained as follows: I) Some kebeles are inaccessible while others are very proximal to urban residence and II) in some area, the social mobilization was poor.

Across age categories, the coverage was 98.3% among children 5–15 years and 87.8% among under-five ones. Similarly, a study conducted in Amhara region revealed that children 6–10 years of age took the treatment two times more likely than children 1–5 years of age [14]. This lower coverage in children 1–5 years of age might be due to fear of side effect among children. This was supported by the qualitative finding. Some FGD discussants reported that they were not comfortable with method of drug dose determination for children. Health works measured child’s height to determine the number of pills, and some of the height measuring rods were inconsistent. As a result of this, there was a fear among parents that some of the children might be taking the amount more than they should. Some participants even mentioned that it is better to give the drug to children based on their age rather than their height.

### Campaign mobilization

Mass drug administration shall be preceded by proper mobilization activities for its successful implementation. In this study, among those who received Zithromax mass treatment, 92.7% were informed about the campaign ahead of time. This result is in agreement with the qualitative finding. According to the respondents, awareness was created in the community before the campaigns in all study Woreda except Raya Alamata. This could heighten their understanding of the benefits of the drug and participation in Zithromax mass drug administration campaign, resulting in increased treatment coverage [20].

During the campaign, health extension workers taught the community on personal hygiene, and to swallow the drug as it prevents from eye disease. Although the information provided is not comprehensive to address all the SAFE strategies for trachoma elimination, it could enhance the community’s acceptance for the mass treatment. This is consistent with the health belief model, which says that knowledge, awareness and attitudes about the diseases positively affect the acceptability of the mass treatment [21].

Here, women development army (43.3%) and health extension workers (32.52%) were the most common source of information for the household heads regarding Zithromax mass drug administration campaign. This finding is in agreement with what was said by the FGD participants and Key informants, that women development armies were the main repository of important information, and their active participation was vital to the successful execution of the Zithromax MDA campaign. The main messages addressed were the date of the campaign and instructing to take the drug with all family members. However, the communities were not sufficiently informed about the benefits and side effects of the intervention and on the need to eat food before coming to the Zithromax MDA campaign.

In contrast, in Awi Zone, Amhara region, community was informed not to drink alcohol and eat enough amount of food before taking the drug. Besides, they were told to drink much water, advised about the benefit of the drug, health educated about personal and environmental sanitation, asked pregnancy status and told about the side effects to increase acceptance and minimize side effect before the campaign [14]. This discrepancy could be explained by the level of pre-campaign planning at the Woreda level. This result is supported by the qualitative finding of this study that in the 2016 Zithromax mass drug administration there was a lack of coordinated mobilization.

From the qualitative finding, mobilizing the whole population at the same day and at the same place for intervention was one of the frequently reported problems. Because, large number of the population would come to the intervention sites that lead to pose a disproportionate burden on the providers to administer and record and leads the service users to grumble for the possible long waiting hours to get the drug administration. Taking a lesson from previous campaigns, in 2016 campaign the community was mobilized at “Gots” level, in a place where convenient to most of the community members.

Despite the majority of the woredas ensure timely distribution of supplies and proper allocation of professionals for every site, there were minor problems. The participants reported that there was a delay in distribution of registration books in Ofla woreda, drug until the third day of the campaign in Raya Azebo, shortage of TTC ointment at the third day of the campaign in Raya Alamata, and shortage of the drug in Seharti Samre.

### Barriers to mass Zithromax administration

Understanding the factors that decrease the acceptability of Zithromax uptake helps identify context-specific challenges. Absence during the campaign (47.5%), mothers misperception of their child as not eligible (16.4%) and lack of awareness about the campaign (9.1%) were bold reasons mentioned by the individuals for non-uptake of Zithromax MDA (Table 4). The context of this survey indicated that the program was not reachable to some segments of the community. This could be because of limited time, poor accessibility of households, lack of available transportation and staff. Refusal and being pregnant were also additional common reasons for not swallowing the drug. This condition showed the importance of health education for the community about the benefits of MDA. Study from Awi zone, Amhara region, reported similar findings: I will consume after meal, too many tablets, seek consent from doctor and lack of awareness[13]. In support of this, in Kenya, the predominant reasons for not receiving Zithromax were lack of awareness, fear of side effects, community misperceptions and malpractices [22].

From the qualitative study, we found widespread barriers in the campaign—primarily driven by frequent occurrence of drug side effects, rumors, transportation constraints, community and leader engagement in else campaigns other than MDA, fasting, shortage of human power and temporarily occurred shortage of supplies. These may attributed to the lower Zithromax MDA coverage in some study settings. Literatures cited that identifying barriers that persist across different health behaviors such as lack of time (due to family, household and occupational responsibilities), access issues (to transport and facilities), entrenched attitudes, restrictions in the physical environment and lack of knowledge can inform the design of tailored interventions for the community[23].

In a study conducted in Northern Tanzanian, the main factors affecting acceptability were local prevention norms (such as the belief on injections rather than oral medicines), perceptions of drugs in general and Zithromax in particular, perceptions of the distribution team’s expertise, witnessing the adverse effects in others, and the timing, quality and quantity of information and its availability[24].

In the present study, the misconceptions and rumors included: it harms reproductive organs, decreases sense of sexuality, and control fertility. In line with this, people in Kenya thought that Zithromax could be a family planning pill [22].

This study has a number of limitations. Firstly, the study was conducted after two months of the Zithromax mass administration campaign that made it prone to recall bias. However, Budge et al, 2016 noted that 12 months’ time interval for a coverage survey is satisfactory [25]. Head of households had reponseded on behalf of their members. Thus, there might be some unavoidable information disparities.

In conclusion, the regional health bureau should arrange logistics such as vehicle, manpower, adequate drug supply, and registration books; provide uniform training for the distributors; plan and inform Woreda ahead of time and plan to have the campaign in a convenient time for the community to facilitate successful implementation of the campaign. The Woreda health officers shall work more in pre-campaign mobilization, and the community shall be informed about the possible side effects and the necessary preconditions. Furthermore, health extension workers shall be involved in the provision of messages focusing on SAFE strategies for trachoma elimination that could enhance the community's acceptance for the mass treatment.

## Supporting information

S1 Checklist STROBE checklist. (DOC)

S1 File Questionnaire. (DOCX)

S1 Table Sampling procedure. (DOCX)

S2 Table Zithromax survey coverage by district and kebele. (DOCX)

## References

[1] Hu VH, Harding-Esch EM, Burton MJ, Bailey RL, Kadimpeul J, Mabey DCW. Epidemiology and control of trachoma: Systematic review. Tropical Medicine and International Health. 2010; Vol. 15 p. 673–91. doi: 10.1111/j.1365-3156.2010.02521.x 20374566 10.1111/j.1365-3156.2010.02521.x PMC3770928

[2] Ngondi J, Reacher M, Matthews F, Brayne C, Emerson P. Trachoma survey methods: A literature review. Bull World Health Organ. 2009;87(2):143–51. doi: 10.2471/BLT.07.046326 19274367 10.2471/BLT.07.046326 PMC2636192

[3] King JD, Teferi T, Cromwell EA, Zerihun M, Ngondi JM, Damte M, et al Prevalence of Trachoma at Sub-District Level in Ethiopia: Determining When to Stop Mass Azithromycin Distribution. PLoS Negl Trop Dis. 2014;8(3):1–10. 10.1371/journal.pntd.0002732 PMC3953063 24625539

[4] Noa Noatina B, Kagmeni G, Mengouo MN, Moungui HC, Tarini A, Zhang Y, et al Prevalence of Trachoma in the Far North Region of Cameroon: Results of a Survey in 27 Health Districts. PLoS Negl Trop Dis. 2013;7(5):1–9. 10.1371/journal.pntd.0002240 PMC3662655 23717703

[5] Light for the World. Vision 2020 mid term sucess and challenges. Trachoma Control in Ethiopia Experiences in the New Millennium [Internet]. 2010. Available from: https://wiki.light-for-the-world.org//images/1/18/Vision&Development2.pdf

[6] Mariotti, Joseph A, Cook and Silvio P. Water and Sanitation-Related Diseases and the Environment: Challenges, Interventions, and Preventive Measures. 2011 175–186 p. 1^st^ edition Wiley-Blackwell

[7] Mariotti SP, Pascolini D, Rose-Nussbaumer J. Trachoma: global magnitude of a preventable cause of blindness. Br J Ophthalmol. 2009;93:563–8. doi: 10.1136/bjo.2008.148494 19098034 10.1136/bjo.2008.148494

[8] Courtright. CM and P. The International Coalition for Trachoma Control (ICTC), Trachoma Action Planning [Internet]. World Health. 2015. Available from: (http://www.trachomacoalition.org/TAPplanning

[9] International Coalition for Trachoma Control. Preferred Practices for Zithromax, Mass Drug Administration [Internet]. International Coalition for Trachoma Control. 2013. Available from:http://www.trachomacoalition.org/sites/default/files/content/resources/files/ICTC_MDAToolkitEN_0.pdf

[10] Burr SE, Milne S, Jafali J, Bojang E, Rajasekhar M, Hart J, et al Mass administration of azithromycin and Streptococcus pneumoniae carriage: cross-sectional surveys in the Gambia. Bull World Health Organ [Internet]. 2014;92(7):490–8. Available from: http://www.scopus.com/inward/record.url?eid=2-s2.0-84903590572&partnerID=tZOtx3y1 doi: 10.2471/BLT.13.133462 25110374 10.2471/BLT.13.133462 PMC4121870

[11] Cumberland P, Edwards T, Hailu G, Harding-Esch E, Andreasen A, Mabey D, et al The impact of community level treatment and preventative interventions on trachoma prevalence in rural Ethiopia. Int J Epidemiol. 2008;37(3):549–58. doi: 10.1093/ije/dyn045 18356196 10.1093/ije/dyn045

[12] World Health Organization. Report of the 18th meeting of the WHO Alliance for the Global Elimination of Trachoma by 2020 [Internet]. Addis Ababa; 2014. Available from: http://apps.who.int/iris/bitstream/10665/163362/1/9789241508681_eng.pdf?ua=

[13] Global health delivery. The Global Trachoma Mapping Project [Internet]. 2016. Available from: www.globalhealthdelivery.org

[14] Zelalem B, Mekonen T. Acceptability of Azithromycin Mass Treatment for Trachoma Elimination in Injibara Town and Adjacent Banja Woreda of Awi Zone, Amhara Region. 2014; Addis Ababa Universilty Online library. www. http://etd.aau.edu.et/handle/123456789/1580

[15] World Health Organization, The London School of Hygiene & Tropical Medicine and the International Trachoma Initiative. Trachoma control : a guide for programme managers [Internet]. 2006. Available from: http://www.who.int/blindness/publications/tcmwho_pbd_get_06_1.pdf

[16] World Health Organization. Zithromax® in the Elimination of Blinding Trachoma: A Program Manager’s Guide. Decatur (Georgia) [Internet]. 2010. Available from: http://www.trachoma.org/sites/default/files/partner-resource/2017-03/itizithromax-managers-guide0.pdf

[17] West SK, Munoz B, Mkocha H, Gaydos CA, Quinn TC. Number of years of annual mass treatment with azithromycin needed to control trachoma in hyper-endemic communities in Tanzania. J Infect Dis. 2011;204(2):268–73. doi: 10.1093/infdis/jir257 21673038 10.1093/infdis/jir257 PMC3114471

[18] Cromwell EA, King JD, McPherson S, Jip FN, Patterson AE, Mosher AW, et al Monitoring of Mass Distribution Interventions for Trachoma in Plateau State, Nigeria. PLoS Negl Trop Dis. 2013;7(1). 10.1371/journal.pntd.0001995 PMC3542118 23326617

[19] Keenan JD, Hotez PJ, Amza A, Stoller NE, Gaynor BD, Porco TC, et al Elimination and Eradication of Neglected Tropical Diseases with Mass Drug Administrations: A Survey of Experts. PLoS Negl Trop Dis. 2013;7(12):1–8. 10.1371/journal.pntd.0002562 PMC3855072 24340111

[20] Amarillo MLE, Belizario VY, Sadiang-Abay JT, Sison SAM, Dayag AMS. Factors associated with the acceptance of mass drug administration for the elimination of lymphatic filariasis in Agusan del Sur, Philippines. Parasites and Vectors. 2008;1(1). 10.1186/1756-3305-1-14 PMC2441609 18505577

[21] Tarkang EE, Zotor FB. Application of the Health Belief Model (HBM) in HIV Prevention: A Literature Review. Science Publishing Group. 2015; Vol. 1 p. 1–8.

[22] Gilbert PC. Coverage and factors influencing uptake of mass drug administration (MDA) using azithromycin for trachoma control in West Pokot District. 2012; London School of Hygiene and Tropical Medicine www.lshtm.ac.uk/library/MSc_CEH/2011-2012/105429.pdf

[23] Kelly S, Martin S, Kuhn I, Cowan A, Brayne C, Lafortune L. Barriers and Facilitators to the Uptake and Maintenance of Healthy Behaviours by People at Mid-Life: A Rapid Systematic Review. PLoS ONE. 2016; 11(1)1–26. 10.1371/journal.pone.0145074 PMC4731386 26815199

[24] Ssemanda EN, Levens J, Mkocha H, Munoz B, West SK. Azithromycin mass treatment for trachoma control: Risk factors for non-participation of children in two treatment rounds. PLoS Negl Trop Dis. 2012;6(3). 10.1371/journal.pntd.0001576 PMC3308937 22448296

[25] Budge PJ, Sognikin E, Akosa A, Mathieu EM, Deming M. Accuracy of Coverage Survey. Recall following an Integrated Mass Drug. Administration for Lymphatic Filariasis, Schistosomiasis, and Soil-Transmitted Helminthiasis. PLoS Negl Trop Dis. 2016; 10(1): e0004358 doi: 10.1371/journal.pntd.0004358 26766287 10.1371/journal.pntd.0004358 PMC4713198

