# Supplementary material for: Coverage, social mobilization and challenges of mass Zithromax administration campaign in South and South East zones of Tigray, Northern Ethiopia: A cross sectional study
Source: PLoS Negl Trop Dis. 2018 Feb 26;12(2):e0006288. doi: 10.1371/journal.pntd.0006288 (PMC5854420; doi:10.1371/journal.pntd.0006288)
Supplement: S1 File — (DOCX) [file pntd.0006288.s002.docx]

## Annex 6: Questionnaire

General instruction: this questionnaire will be used to assess the post Zithromax MDA coverage in South east and southern zones of Tigray, Northern Ethiopia

**Questionnaire Code**________________________

**Zone: __________________Woreda:_________________Tabia/Kebele: ________________ Date of visit: _______________**

**Section I: Demographic and socioeconomic profile of the household heads**

| **S.N.** | **Item/Question** | **Response** | **Remark** |
| --- | --- | --- | --- |
| 101 | Sex of respondent | 1. Male 2. Female |  |
| 102 | Age(in years) | _______ |  |
| 103 | Residency | 1. Urban 2. Rural |  |
| 104 | How long have you been living in the community? | 1. =< 6 months 2. > 6 months |  |
| 105 | Religion | 1. Orthodox 2. Muslim 3. Protestant 4. Others |  |
| 106 | Marital status | 1. Single 2. Married 3. Divorced   4.Widowed  5. Other, specify------------------ |  |
| 107 | Educational status | 1. Unable to read and write 2. Able to read and write 3. 1-4 Grade complete 4. 5-8 Grade complete 5. 9-12 grade complete 6. College/diploma complete 7. College/degree complete |  |
| 108 | Occupation | 1. Student 2. Merchant 3. Farmer 4. Government employee 5. NGO 6. House wife 7. Daily Worker 8. Jobless 9. Others |  |
| 109 | Family monthly income | ____________________(Birr) |  |
| 110 | Family m-onthly expenditure | ____________________(Birr) |  |
|  | **Section II: Questions related to azithromycin mass treatment for the household head** | | |
| 201 | Did you take Zithromax? show them sample drug | 1. Yes 2. No | **If no, go to Q 205** |
| 202 | If “Yes” to Q 201 when was that? | 1. June 2015 2. May, 2016 3. Both |  |
| 203 | Do house member know the disease treated against? | 1. Yes 2. No |  |
| 204 | If yes, what it is? | _______________ |  |
| 205 | If “NO”, to # 201, what was the reason why you didn’t get Azithromycin treatment? | 1. Absent 2. Refusal 3. House not visited 4. Not aware/didn’t hear about campaign 5. Pregnant 6. Breast feeding 7. At school 8. Other(specify)-------------- |  |
| 206 | Reason for Absence(if response to Q 205 is 1: Absent) | 1. School 2. Market 3. Church/Mosque 4. Out with animals 5. Getting water/wood 6. Farming 7. Other(specify)------------- |  |
| 207 | Reason for Refusal(if response to Q 205 is 2: Refusal) | 1. Religious beliefs 2. HH Member was sick 3. Drug was not safe 4. Not happy with the health team 5. Other(Specify)----------- |  |
| 208 | Have the parents been informed about the campaign before the Azithromycin treatment supplying team has visited the house? | 1. Yes 2. No | **If no, go to Q 210** |
| 209 | If the response to Q 208 is yes, how did they get the information | 1. Women development army 2. Health extension worker 3. Other health professionals 4. Community leaders 5. Kebele leaders 6. Religious leaders 7. Social mobilizers/Public criers 8. Radio 9. TV 10. Others, specify------------- |  |
| 210 | Did you get health education from HCW about Azithromycin? | 1. Yes 2. No |  |
| 211 | If yes, what information were you delivered? (Probe him/her) | ---------------------------------------------------------------------------------------- |  |

**Section III: Questions related to azithromycin mass treatment for household head about his**

**Family members and for adults**

| **S. No** | **Question** | **House Hold Members(______________#)** | | | | | | | | | | | |
| --- | --- | --- | --- | --- | --- | --- | --- | --- | --- | --- | --- | --- | --- |
|  |  | Wife/husband | 01 | 02 | 03 | 04 | 05 | 06 | 07 | 08 | 09 | 10 | 11 |
| 301 | Sex |  |  |  |  |  |  |  |  |  |  |  |  |
| 302 | Age in years |  |  |  |  |  |  |  |  |  |  |  |  |
| 303 | Occupation |  |  |  |  |  |  |  |  |  |  |  |  |
| 304 | Marital status |  |  |  |  |  |  |  |  |  |  |  |  |
| 305 | Occupation |  |  |  |  |  |  |  |  |  |  |  |  |
| 306 | Did s/he take Zithromax in last campaign (May/June 2016)? (Yes/no): show them sample drug |  |  |  |  |  |  |  |  |  |  |  |  |
| 307 | If “NO”, why? |  |  |  |  |  |  |  |  |  |  |  |  |

**Sex:** (1= Male, 2= Female)

**Occupation:** (1**=**Student, 2= Merchant, 3=Farmer, 4=Government employee, 5= House wife, 6=NGO, 7=Jobless, 8= others)

**Marital status:** (1= Single, 2= Married, 3=Divorced, 4=.Widowed)

**Educational Status**: (1=Unable to read and write; 2= Able to read and write; 3= 1-4 Grade complete, 4= 5-8 Grade complete; 5= 9-12 grade complete; 6 =College/diploma complete; 7= College/degree complete)

**Reasons:** (1= absent; 2= Refusal; 3= House not visited; 4= Not aware/didn’t hear about campaign; 5= Pregnant; 6= Breast feeding; 7= at school; 8= other (specify) --------------)

***End of Interview!***
**THANK YOU FOR YOUR INFORMATION AND TIME**

**FGD Guide**

1. Is trachoma a common problem in the community?
2. What are the causes of trachoma?
3. Who are the most highly affected people by trachoma?
4. How do we prevent trachoma?
5. How many times was the azithromycin mass treatment program given in your kebele?
6. How is the acceptability of Azithromycin drug treatment by the community?
   1. If low- Why?
   2. If it is high – How/Why is this?
7. How was the azithromycin distributed?
8. Was the azithromycin distribution method acceptable by the community?(house to house, HF, market, etc)
9. What is the community perception and experience about azithromycin side effect?
10. Are there persons who didn’t participate in any of the programs? Why?
11. Why do people refuse to take the drug/to participate in the program?
12. Was health education given before the MDA?
13. What is the opinion of the community to azithromycin mass treatment program?
14. For what purpose did the community use this drug other than trachoma control?
15. Who provided the drug?
16. How is the interaction of the community and treatment providers?

**In-depth Interview Guide**

**Section I: Semi structured questions for key informant interview for Woreda Health officers**

**1.** How was the azithromycin mass treatment program going on?

**2.** How is the mass azithromycin treatment coverage for each campaign?

**3.** How was the communities’ acceptability of the program?

**4.** What challenges from the community were reported during the drug distribution?

**Section II: Semi structured ques-tions for key informant interview to health extension**

**Workers**

1. What practical challenges had you faced during the drug administration?

2. What rumours had you heard from the community about the drug?

3. What were the mostly raised reasons by persons who refused to take azithromycin mass treatment?

4. What factors do you think that affects the drug treatment acceptability by the community?

5. What solutions were taken for persons who were refused to take the drug?

6. Had you noticed any health problem in persons after the drug administration? If yes, what were the health problems you encounter?

***End of Interview!***
**THANK YOU FOR YOUR INFORMATION AND TIME**
