# Supplementary material for: Coverage, social mobilization and challenges of mass Zithromax administration campaign in South and South East zones of Tigray, Northern Ethiopia: A cross sectional study
Source: PLoS Negl Trop Dis. 2018 Feb 26;12(2):e0006288. doi: 10.1371/journal.pntd.0006288 (PMC5854420; doi:10.1371/journal.pntd.0006288)
Supplement: S1 Table — (DOCX) [file pntd.0006288.s003.docx]

**Annex.**

**Annex1. Table showing Sampling procedure in the nine MDA campaign Woreda in South east and Southern zones of Tigray, Ethiopia, 2016**

| **Zone** | **Woreda** | **Selected Kebeles** | **Total number of households in each Kebele** | **Number of sampled households** |
| --- | --- | --- | --- | --- |
| **South East Tigray** | **Hintalowajrat** | 1. Adikeyih | 1,747 | 21 |
|  |  | 1. Hintalo | 1,408 | 13 |
|  |  | 1. Fireweyni (Hiwane) | 1,671 | 17 |
|  |  | 1. Bahri tseba | 2,190 | 28 |
|  |  | 1. Hareko | 2,326 | 27 |
|  | **Samre Seharti** | 1. .May Tekli | 1875 | 22 |
|  |  | 1. Waza | 881 | 26 |
|  |  | 1. Samre | 1250 | 23 |
|  |  | 1. Adi Abso | 1760 | 22 |
|  | **Enderta** | 1. May Anmbessa | 1,328 | 15 |
|  |  | 1. Felege Selam | 1,725 | 26 |
|  |  | 1. Romanat | 2,085 | 25 |
|  |  | 1. Didba | 2,169 | 19 |
|  | **Degua Temben** | 1.Arebaye | 784 | 15 |
|  |  | 2.Melfa | 1,085 | 20 |
|  |  | 3.Simret | 1,488 | 28 |
|  |  | 4.Limeat | 1,232 | 21 |
| **South Tigray** | **Ofla** | 1.Fikrewolda | 936 | 17 |
|  |  | 2.Hashege | 2,123 | 40 |
|  |  | 3.Fala | 1,282 | 26 |
|  |  | 4.Hugumbrda | 1,700 | 48 |
|  | **Endamokeni** | 1.Emba-hasti | 954 | 18 |
|  |  | 2. Senay | 1041 | 24 |
|  |  | 3.Tsibet | 1398 | 27 |
|  |  | 4.Mekan | 1552 | 29 |
|  | **Raya Azebo** | 1.Mekoni | 3,799 | 52 |
|  |  | 2.Hawelti | 2,715 | 37 |
|  |  | 3.Kukfto | 2,448 | 27 |
|  |  | 4.Mechare | 2,439 | 37 |
|  | **Emba – Alaje** | 1.Sesat | 1,932 | 40 |
|  |  | 2.Mayleham | 1,409 | 23 |
|  |  | 3.Bora | 1,171 | 27 |
|  |  | 4.Betmayra | 1,430 | 27 |
|  | **Raya Alamata** | 1.Tumuga | 1,469 | 23 |
|  |  | 2.Tao | 1,880 | 22 |
|  |  | 3.Garjale | 1,675 | 19 |
| **Total** | **9 Woredas** | **36 kebles** | **60,357** | **931** HHs |
