# Supplementary material for: Coverage, social mobilization and challenges of mass Zithromax administration campaign in South and South East zones of Tigray, Northern Ethiopia: A cross sectional study
Source: PLoS Negl Trop Dis. 2018 Feb 26;12(2):e0006288. doi: 10.1371/journal.pntd.0006288 (PMC5854420; doi:10.1371/journal.pntd.0006288)
Supplement: S2 Table — (DOCX) [file pntd.0006288.s004.docx]

**Annex2: Table showing Zithromax MDA Coverage at Woreda level**

| **S.N** | **WOREDA** | **Number of Households** | **Number of eligible individuals** | **Number of individuals swallowed Zithromax and proportion** |
| --- | --- | --- | --- | --- |
| 1 | **Seharti_Samre** | 93 | 341 | 307 (90.0%) |
| 2 | **Enderta** | 85 | 346 | 318 (91.9%) |
| 3 | **Degua_Temben** | 84 | 373 | 346 (92.8%) |
| 4 | **Raya_Azebo** | 153 | 581 | 539 (92.8%) |
| 5 | **Ofla** | 131 | 526 | 496 (94.3%) |
| 6 | **Raya_Alamata** | 64 | 244 | 222 (91.0%) |
| 7 | **Hintalowajrat** | 106 | 436 | 421 (96.6%) |
| 8 | **Emba_Alaje** | 117 | 461 | 416 (90.2%) |
| 9 | **Endamokeni** | 98 | 433 | 424 (97.9%) |
|  | **Total** | **931** | **3741** | **3489 (93.3%)** |

**Annex3: Table that show Zithromax coverage at Kebele level, South east and Southern Tigray, Ethiopia, 2016**

| **S.N** | **Selected Kebele** | **Number of Households selected** | **Number of eligible individuals** | **Number of individuals swallowed Zithromax and proportion** |
| --- | --- | --- | --- | --- |
| 1 | Adi_Abso | 22 | 73 | 63(86.3% |
| 2 | Adi_Keyih | 21 | 72 | 69(95.8%) |
| 3 | Arebaye | 15 | 72 | 70 (97.2%) |
| 4 | Bahri_tseba | 28 | 100 | 95 (95.0%) |
| 5 | Betmayra | 27 | 98 | 86 (87.8%) |
| 6 | Bora | 27 | 97 | 78 (80.4%) |
| 7 | Didba | 19 | 65 | 49 (75.4%) |
| 8 | Emba_Hasti | 18 | 72 | 71 (98.6%) |
| 9 | Fala | 26 | 108 | 100(92.6%) |
| 10 | Felege_Selam | 26 | 114 | 112 (98.2%) |
| 11 | Fikrewolda | 17 | 75 | 73 (97.3%) |
| 12 | Garjale | 19 | 75 | 68(90.7%) |
| 13 | Hareko | 27 | 109 | 105 (96.3%) |
| 14 | Hashege | 40 | 155 | 142 (91.6%) |
| 15 | Hawelti | 37 | 150 | 145 (96.7%) |
| 16 | Hintalo | 13 | 48 | 46 (95.8%) |
| 17 | Hiwane | 17 | 108 | 107 (99.1%) |
| 18 | Hugumbrda | 48 | 187 | 180 (96.3%) |
| 19 | Kukfto | 27 | 88 | 87 (98.9%) |
| 20 | Limeat | 21 | 83 | 74 (89.2%) |
| 21 | May_Ambesa | 15 | 61 | 56 (91.8%) |
| 22 | May_Tekli | 22 | 77 | 69 (89.6%) |
| 23 | Mayleham | 23 | 97 | 94 (96.9%) |
| 24 | Mechare | 37 | 131 | 119 (90.8%) |
| 25 | Mekan | 29 | 110 | 104 (94.5%) |
| 26 | Mekoni | 52 | 213 | 189 (88.7%) |
| 27 | Melfa | 20 | 64 | 58 (90.6%) |
| 28 | Romanat | 25 | 106 | 101 (95.3%) |
| 29 | Samre | 23 | 84 | 74 (88.1%) |
| 30 | Senay | 24 | 123 | 123 (100.0%) |
| 31 | Shesat | 40 | 169 | 158 (93.5%) |
| 32 | Simret | 28 | 154 | 144 (93.5%) |
| 33 | Tao | 22 | 100 | 93 (93.0%) |
| 34 | Tsibet | 27 | 128 | 126 (98.4%) |
| 35 | Tumuga | 23 | 69 | 61 (88.4%) |
| 36 | Waza | 26 | 106 | 100 (94.3%) |
|  | **Total** | **931** | **3741(100.0%)** | **3489(93.3%)** |
